# Strontium mineralization of shark vertebrae

**DOI:** 10.1038/srep29698

**Published:** 2016-07-18

**Authors:** Vincent Raoult, Victor M. Peddemors, David Zahra, Nicholas Howell, Daryl L. Howard, Martin D. de Jonge, Jane E. Williamson

**Affiliations:** 1 Department of Biological Sciences, Macquarie University, Sydney NSW 2109, Australia; 2 Fisheries NSW, Sydney Institute of Marine Science, 19 Chowder Bay Road, Mosman NSW 2088, Australia; 3 ANSTO Life Sciences, Lucas Heights, NSW 2234, Australia; 4 Australian Synchrotron, 800 Blackburn Road, Clayton, Victoria 3168, Australia

## Abstract

Determining the age of sharks using vertebral banding is a vital component of management, but the causes of banding are not fully understood. Traditional shark ageing is based on fish otolith ageing methods where growth bands are assumed to result from varied seasonal calcification rates. Here we investigate these assumptions by mapping elemental distribution within the growth bands of vertebrae from six species of sharks representing four different taxonomic orders using scanning x-ray fluorescence microscopy. Traditional visual growth bands, determined with light microscopy, were more closely correlated to strontium than calcium in all species tested. Elemental distributions suggest that vertebral strontium bands may be related to environmental variations in salinity. These results highlight the requirement for a better understanding of shark movements, and their influence on vertebral development, if confidence in age estimates is to be improved. Analysis of shark vertebrae using similar strontium-focused elemental techniques, once validated for a given species, may allow more successful estimations of age on individuals with few or no visible vertebral bands.

Humanity’s growing demand for protein has led to substantial pressure on both terrestrial and oceanic ecosystems. Harvesting of large predatory fishes by industrial fishing techniques has lowered populations to a fraction of their historic numbers in some areas^1^,^2^, with some species such as tuna or grouper more depleted than others^3^,^4^. Sharks are especially vulnerable to fishing due to their low reproductive rates and delayed maturity^5^,^6^,^7^. Their disappearance has negative effects on ecosystems^8^, and considerable resources have been allocated to their management and conservation. Basic information about life history parameters such as reproduction, diet, and age structure are often lacking, however, making assessment and management of shark stocks difficult.

Ageing sharks is problematic due to the large numbers of samples required, which are often difficult to obtain^9^. Ageing also requires cross-validation, and a lack of calcified structures in the many deeper-water species has contributed to discrepancies between growth and age estimates^9^. Inaccurate estimates can lead to poor predictions of population growth and lead to overfishing^10^ or, conversely, under-exploitation of resources. Typically, shark ageing has been based on a modification of fish otolith ageing but uses vertebral bands instead of otolith rings^9^, which are counted as a proxy for yearly growth patterns under the assumption that banding patterns result from changes in calcification rates over time in a manner similar to that of fish^11^,^12^,^13^. Although the seasonality of these bands has been validated for many species^14^,^15^, the processes that determine the formation of these bands are not fully understood. Moreover, published methods of shark ageing vary substantially because different preparation methods must be tested to determine the most effective technique for each newly tested species^9^. Different ageing methods and subjective differences in readability make comparative studies problematic^11^,^16^,^17^.

To investigate the elemental composition of vertebral bands, and by association, the factors that may influence their deposition, elemental distribution within vertebrae was assessed in five diverse species of sharks using Scanning X-ray Fluorescence Microscopy (SXFM)^18^. Assessments of SXFM images were compared to traditional microscope ageing^9^ to determine their use as an alternate ageing technique.

## Results

**Figure 1:**
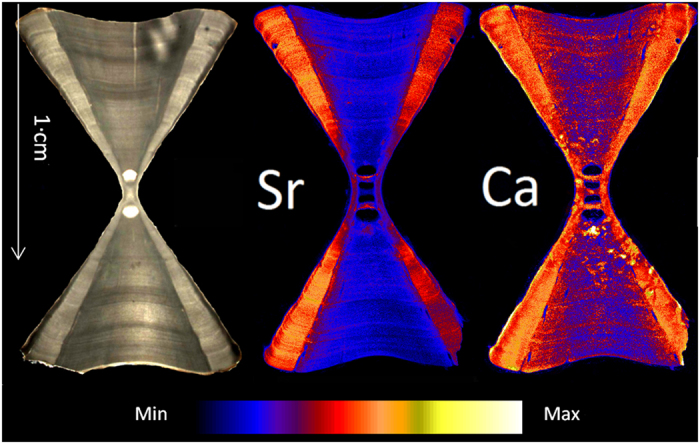
Images of light microscopy (left side) and SXFM (centre, right side) at different elemental spectra (in ppm) of unstained sagittal sections of a 1.7 m female Smooth Hammerhead (*Sphyrna zygaena*) at 25 μm resolution. Banding studies usually focus on the *corpus calcareum*, the denser area on the outside of the vertebrae.

**Figure 2:**
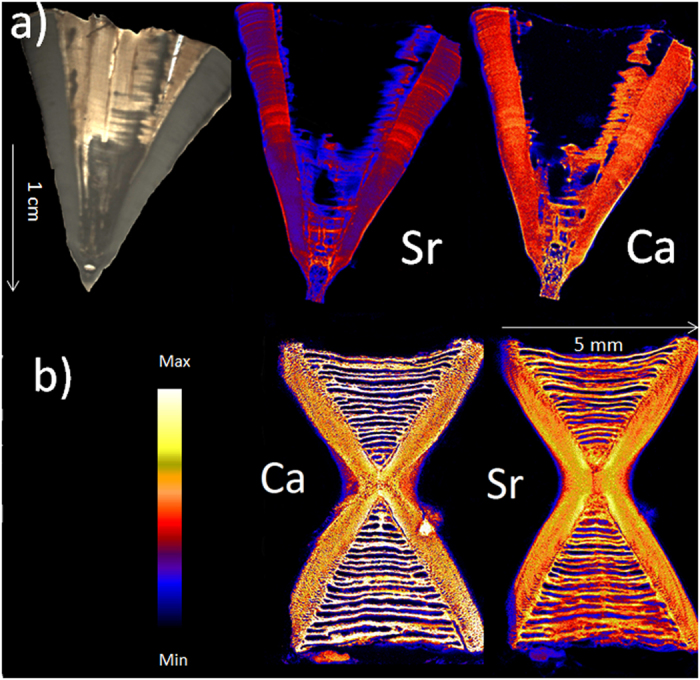
Images of light microscopy (top left) and SXFM (all others) at different elemental spectrums (in ppm) of sectioned vertebrae of (**a**) a 2.7 m White Shark (*Charcharodon carcharias*) vertebra and (**b**) an 0.9 m Eastern angel shark (*Squatina albipunctata)* at 25 and 15 μm resolution, respectively. Light microscopy of angel shark not shown due to lack of visible growth bands.

**Figure 3:**
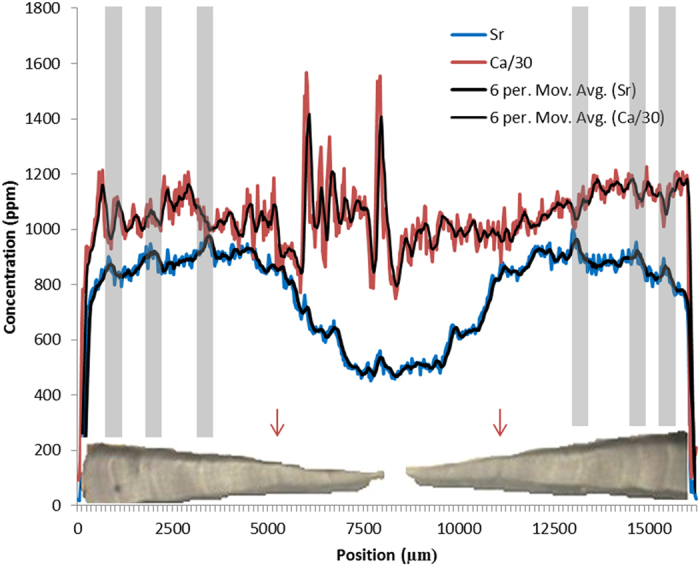
SXFM calcium and strontium concentrations of the *corpus calcarum* from the dorsal to ventral edge of the 1.7 m Smooth Hammerhead (*Sphyrna zygaena*) shown in Fig. 1. Calcium concentrations were reduced by a ratio of 30 to make them comparable to strontium concentrations. Six point moving averages were added to reduce noise. Birth marks are at roughly 5000 and 12000 μm (indicated with red arrows). Data are compared to a flattened horizontal microscope image of the *corpus calcarum* of the vertebra with marked ‘traditional’ growth bands (grey bands that are lined up with the opaque winter bands throughout the thickness of the vertebra for comparison with strontium and calcium). Note how strontium peaks are consistent on both sides of the vertebra, while calcium concentrations are not.

**Figure 4:**
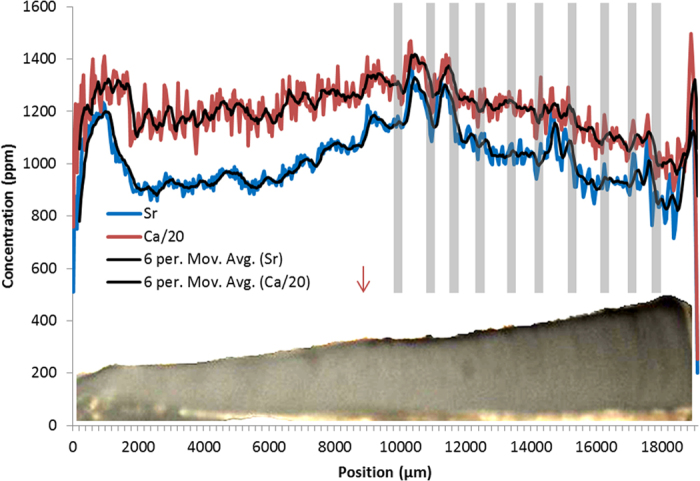
SXFM calcium and strontium concentrations of the *corpus calcarum* from the dorsal to central edge of a 2.7 m female White Shark (*Charcharodon carcharias*). Calcium concentrations were reduced by a ratio of 20 to make them comparable to strontium concentrations. Six point moving averages were added to reduce noise. Birth mark is at 9000 μm (indicated with red arrow). Data were compared to a flattened horizontal microscope image of the *corpus calcarum*, with marked ‘traditional’ growth bands (grey bands that are lined up with the opaque winter bands throughout the thickness of the vertebra for comparison with strontium and calcium).

**Figure 5:**
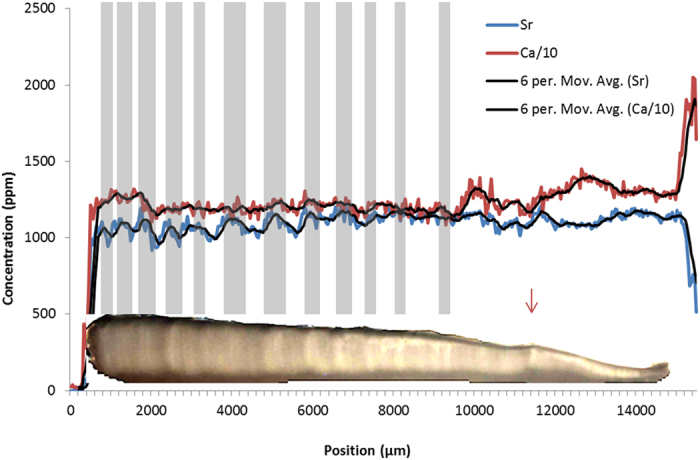
SXFM calcium and strontium concentrations of the *corpus calcarum* from the dorsal to ventral edge of a 2.4 m female Spinner Shark (*Carcharhinus brevipinna*). Calcium concentrations were reduced by a ratio of 10 to make them comparable to strontium concentrations. Six point moving averages were added to reduce noise. Birth mark is roughly at 11500 μm (indicated with red arrow). Data are compared to a flattened microscope image of the *corpus calcarum* of the vertebra with marked ‘traditional’ growth bands (grey bands that are lined up with the opaque winter bands throughout the thickness of the vertebra for comparison with strontium and calcium).

**Figure 6:**
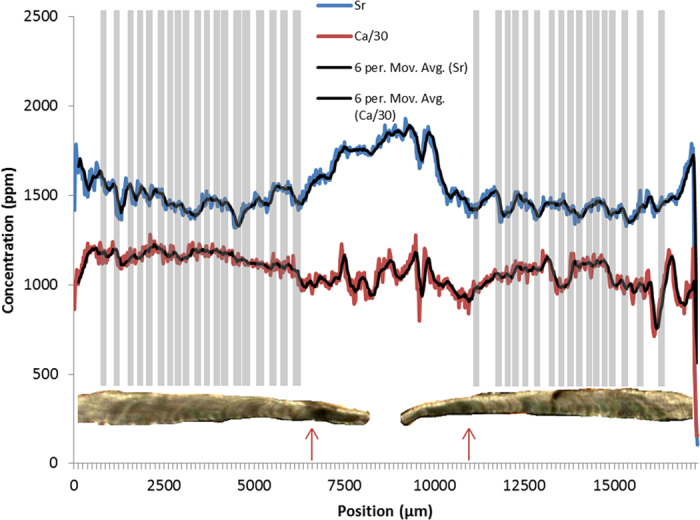
SXFM calcium and strontium concentrations of the *corpus calcarum* from the ventral to dorsal edge of a 1.15 m female Port Jackson Shark (*Heterodontus portusjacksoni*). Calcium concentrations were reduced by a ratio of 30 to make them comparable to strontium concentrations. 6 point moving averages were added to reduce noise. Birth marks are roughly at 7000 and 11000 μm (indicated with red arrow). Data are compared to a flattened microscope image of the *corpus calcarum* of the vertebra.

**Figure 7:**
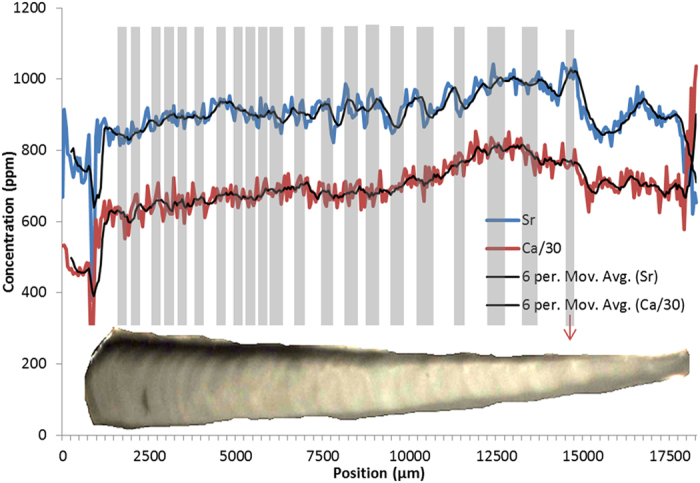
SXFM calcium and strontium concentrations of the *corpus calcarum* from the dorsal to ventral edge of a 2.8 m female Dusky Shark (*Carcharhinus obscurus*). Calcium concentrations were reduced by a ratio of 30 to make them comparable to strontium concentrations. 6 point moving averages were added to reduce noise. Birth mark is roughly at 14500 μm (indicated with red arrow). Data are compared to a flattened microscope image of the *corpus calcarum* of the vertebra with marked ‘traditional’ growth bands (grey bands that are lined up with the opaque winter bands throughout the thickness of the vertebra for comparison with strontium and calcium).

**Figure 8:**
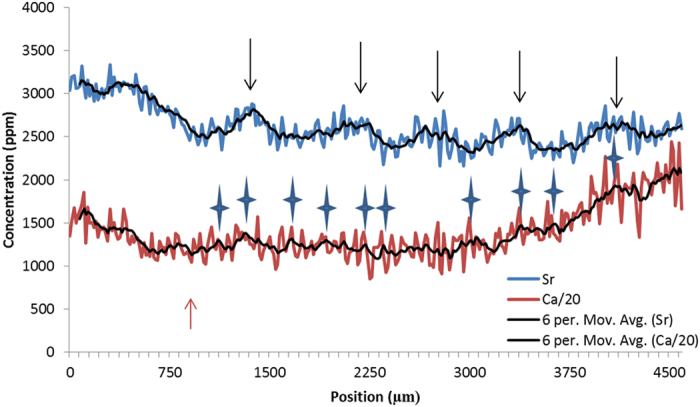
SXFM calcium and strontium concentrations of the *corpus calcareum* from the dorsal to ventral edge of an 839 mm male Eastern angel shark (*Squatina albipunctata*). Calcium concentrations were reduced by a ratio of 30 to make them comparable to strontium concentrations. 6 point moving averages were added to reduce noise. Strontium peaks are marked with black arrows and calcium peaks with blue stars. Birth mark is roughly at 1000 μm (indicated with red arrow). Image of light microscopy not shown as no bands were visible on the *corpus calcareum* in this study or in others^52^,^53^.

**Figure 9:**
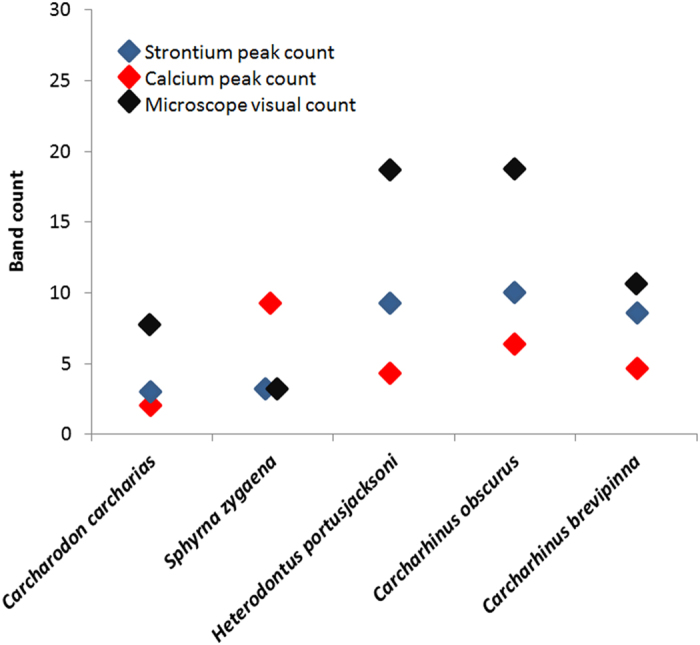
Counts of strontium and calcium peaks, compared to traditional microscope ageing length for (left to right): White Shark (*Carcharodon carcharias)*, Smooth Hammerhead (*Sphyrna zygaena*), Port Jackson Shark (*Heterodontus portjacksoni*), Dusky Shark (*Carcharhinus obscurus*) and Spinner Shark (*Carcharhinus brevipenna*).

Calcium and strontium maps showed clear banding within vertebrae of all species. However, strontium bands were consistently more visible than calcium bands (Figs 1 and 2). Strontium peak counts agreed with, or were slightly under, counts obtained microscopically, while calcium peak counts could be both less than or greater than microscope band counts (Figs 3, 4, 5, 6, 7, 8). In all cases, counts of strontium peaks corresponded more closely to banding counts determined by light microscopy than to counts of calcium peaks (Fig. 9). Neither strontium concentrations (df = 4, F = 6.7, R^2^ = 0.69, p = 0.08) nor calcium concentrations (df = 4, F = 0.43, R^2^ = 0.12, p = 0.55) significantly correlated with light microscopy band counts. However, the correlation coefficient (R^2^) of the strontium to light microscopy relationship was much higher than the calcium relationship (0.69 vs 0.12).

## Discussion

In some species, shark vertebral growth banding may be more closely related to strontium absorption rather than to calcification. Strontium and calcium peaks did not correlate. Rather, strontium peak numbers were more closely related to counts obtained using light microscopy than were calcium peaks. It is possible that the small sample size in this study prevented the establishment of significant statistical relationships. However, strontium data had a substantially stronger relationship with traditional counts via light microscopy than the calcium data from the same samples. If further individuals were analysed in a similar fashion, it is likely that a statistically significant correlation between strontium and light microscopy bands would exist. In addition, the peaks on the dorsal and ventral face of the vertebrae had symmetrical shapes and positioning (peaks were equidistant on dorsal and ventral sides), whereas calcium peaks were asymmetrical. Since growth deposition is physiological, symmetrical patterns are expected in the banding.

### Origins of strontium variation

Strontium is absorbed during otolith mineralization in fish, often replacing calcium^19^, and otolith absorption rates are dependent on environmental concentrations of the element^20^. Strontium is preferentially absorbed relative to other trace elements present in the marine environment (e.g., magnesium, barium)^21^, whereas calcium is absorbed at a relatively constant rate regardless of its environmental availability^20^. Though the processes that govern the absorption of strontium in fish are still debated, the model that best explains these contradicting results is that preferential strontium absorption is linked to the rate of proteinacious matrix formation^21^. Incorporation of strontium into fish otoliths is not immediate: changes in concentrations of ambient water strontium have effects on mineralized concentrations but only after ten days, and sometimes does not become visible until sixty days after exposure^22^. Despite the apparent role of strontium in fish development, the physiological role of this element is still unknown^23^.

Strontium does modulate bone metabolism in mammals: higher strontium concentrations increase bone mass^24^ or prevent bone loss^25^. In humans, high-strontium-content derivatives significantly decrease vertebral and non-vertebral fracture risk^26^,^27^, offering promise as an anti-osteoporitic and anti-osteoarthritic in medicine. Recent studies that have tried to determine how strontium affects mammalian bone growth have found that nearly 50% of available strontium is incorporated into calcium hydroxyapatite or absorbed into collagen^28^. Strontium effects on mammalian cartilage are less well studied, but evidence suggests that increased strontium levels promote cartilage matrix formation^29^. While mammalian and chondrichthyan bone/cartilage development are unlikely to be completely analogous, mammalian skeletal elements are deposited on cartilaginous models, and the mineral fraction of elasmobranch vertebrae is similar in composition to that of mammals^30^. It is, therefore, probable that strontium also affects the resorption/formation of elasmobranch cartilage and vertebral bands in a similar fashion as in mammalian bones.

In this study, strontium peaks sometimes corresponded with thinner, opaque regions known as winter bands^31^. These areas precede areas of higher growth that have lower strontium concentrations. It is possible that the accumulation of strontium allows increased bone development in subsequent months, resulting in thinner, high strontium ‘winter’ bands and thicker, low strontium ‘summer’ bands in the sharks. The opacity of vertebral bands observed in this study may also be due to a denser cartilage matrix, similar to those detected in mammals^29^.

Previous research assumed that shark growth bands were caused by environmental variables such as temperature and salinity, resulting from changes in environmental conditions^32^. Spectrometry has shown that temperature has no effect on strontium concentrations in elasmobranch vertebrae, although increased temperatures increase rates of vertebral growth in these species^33^. Otolith strontium/calcium ratios in teleost fish are positively linked to environmental strontium concentrations^34^, and are directly related to salinity in some species^35^,^36^, but are usually unrelated to temperature^35^,^37^. Since salinity varies seasonally^38^ and increases with depth^39^, vertebral growth bands may be partially caused by seasonal or depth salinity gradients rather than by changes in metabolic rates resulting from seasonal variations in temperature. This also raises the possibility of determining the salinity of areas visited by the shark, as estuarine movements have been estimated from chemical signatures of teleost otoliths^40^,^41^,^42^. Similar conclusions were obtained from analyses of mass spectrometry on shark vertebral composition, where lower strontium/barium ratios in *Carcharhinus leucas* were suggested to be related to movements into estuarine waters^43^. Smalltooth sawfish (*Pristis pectinata*) also show strontium variations in relation to changes in salinity^44^. One of our model species, *Squatina albipunctata*, is thought to come inshore every year to pup or mate, which may explain the changes in strontium concentrations we observed. While correlations between strontium and visual growth bands for *Heterodontus portjacksoni* were weaker at younger ages, they more closely match strontium peaks in the animals matured. Port Jackson Sharks (*H. portjacksoni*) congregate annually for reproduction in estuaries once sexually mature^45^. It is possible that this post-maturity yearly migratory pattern may be reflected in the increased correlation between visual bands in later years. *Carcharodon carcharias* movement patterns in south-east Australia are not well understood, but tagging of young individuals (1.8–3.6 metres in total length) suggests that they undertake sporadic, large-scale movements^46^, which are consistent with our findings. Future studies should examine the potential drivers of vertebral banding and their links with environmental variables, with a particular focus on the interactions between temperature, salinity, and strontium availability.

### Strontium maps

Shark ageing can be affected by observer bias^9^, however, the use of strontium maps can decrease subjectivity. In conjunction with traditional ageing methods, strontium mapping may increase the accuracy of measurements and/or allow ageing where it was previously unsuccessful (e.g. *Squatina sp*.), but only when species-specific seasonal movement patterns are understood and/or the technique has been validated with individuals of known age. Round stingrays (*Urobatis halleri*) have vertebral strontium concentrations that correlate well with growth bands^47^, and they also have seasonal movement patterns. Strontium deposition needs to be further investigated to determine whether it is more reliable for band analysis than methods that analyse calcium concentrations, and how/why strontium is absorbed. If strontium is a better banding indicator than calcium, it would explain why chemical stains historically used in shark ageing (alizarin red, silver nitrate) have varying results, since they are indicators for calcium, strontium, magnesium and iron. The use of directed strontium reporters may, therefore, produce better results.

The effectiveness of strontium maps as an ageing tool could be validated as a technique by using age-validated samples for each species (from tagging programs, bomb-radiocarbon dating specimens, or marking captive sharks with oxytetracycline), but may only be effective if species’ migration patterns are known. Movement patterns of elasmobranchs can change with ontogeny^48^, can be sex-linked^49^, and may show a high degree of intraspecific variation^46^. Consequently, reliable ageing of species using strontium mapping requires highly predictable migratory patterns that occur at a population level, and at consistent intervals, and with known ontogenetic variations. Some species of sharks do not fit these criteria. For example, *C. carcharias* (White Shark) on the east coast of Australia can disperse north and south, with individual preferences^46^. Consequently, strontium maps could not be used to determine the age of *C. carcharias* off Australia. This may explain the disparity between visual band counts and strontium peak counts for the *C. carcharias* sample in this study. However, generalisations should not be made a species level. Female *C. carcharias* in the northeast Pacific have two-year migration intervals between aggregation areas, and male sharks return annually^49^,^50^. Due to their highly reliable movement patterns, strontium maps could be used to determine the age of *C. carcharias* from the northeast Pacific if the sex of the shark was known. Strontium mapping may thus be useful for elasmobranch vertebrae sourced from populations with known seasonal migrations that cannot be aged using traditional techniques. If population-specific information was unavailable, strontium maps would still be useful to infer movement patterns across salinity gradients, but not for aging.

### SXFM

SXFM allowed novel, high resolution, and rapid analysis of the elemental composition of shark vertebrae. Nevertheless, future use of SXFM for analysis of shark vertebrae could be improved. A moving average, used to reduce noise, reduced the effective spatial resolution to six times the sampling interval (between 90 μm and 150 μm). The lowered resolution would explain why the oldest shark in this study did not display strontium peaks that corresponded to visual bands after nine years of age (Fig. 7). Scanning with a finer sampling interval, longer dwell (time spent exposing the area) and a wider sampling band are all feasible using the SXFM, but those measurement parameters would have increased scanning time. Using growth band counts to age older individuals is known to lead to age under-estimation due to the greater proximity of growth bands^47^,^51^, and so higher resolution scans – of order 1 μm – would be of great interest. While variations in specimen thickness and surface topography can confuse the clear identification of banding, the measurements reported in this study are relatively free of such artefacts. We are currently developing additional methods to reduce the impact of measurement bias that will improve the resolution of this approach.

In summary, the presence of strontium bands in shark vertebrae supports new, comparative, alternative ageing methods once validation studies have been conducted. More precise and better-understood shark ageing will lead to more accurate depictions of life histories and population dynamics, supporting more effective management of shark populations and associated ecosystems exploited by humans.

## Materials and Methods

### Collection of sharks

Six species of shark were analysed in this study: *Sphyrna zygaena* (Smooth Hammerhead)*, Charcharodon carcharias* (White Shark)*, Carcharhinus brevipinna* (Spinner Shark)*, Heterodontus portusjacksoni* (Port Jackson Shark)*, Carcharhinus obscurus* (Dusky Shark), and *Squatina albipunctata* (Eastern Angel shark; this genus is known to have no visible growth bands in the corpus calcarum^52^,^53^ and little known of their biology^54^). Sharks were caught by the New South Wales (NSW) Shark Meshing (Bather Protection) Program, with the exception of *S. albipunctata*, which was caught by a commercial fishing trawler near Sydney, Australia. NSW shark nets are set during the summer period along the Newcastle to Wollongong region in New South Wales, Australia^55^. The Animal Ethics Committee at Macquarie University agreed that ethics approval was not required for this study. Tissue samples were retrieved from animals caught by commercial fisheries for sale in local markets and/or the NSW Shark Meshing (bather protection) program, which occasionally catches and kills local wildlife, and thus sharks were obtained for purposes other than research.

### Laboratory preparation of vertebral sections

Vertebral samples were collected by removing the first cervical vertebra from each carcass and freezing it (−20 °C) in a sealed plastic bag until preparation. During preparation, each vertebra was thawed to room temperature, and then manually cleaned of debris with a sterile scalpel before being sectioned. To avoid possible contamination no chemical agents (e.g., sodium hypochlorite, ethanol) were used during cleaning, and the decision was made to avoid using other cleaning techniques (i.e., sonication) because the SXFM is less prone to surface contamination than other elemental techniques. Vertebrae were sectioned using an Isomet circular saw with a single 0.1 mm-increment adjustable diamond-edged blade. Sections were cut dorso-ventrically through the centre of each vertebra. Cuts were 0.6 mm in thickness or thinner, dependent on the degree calcification (more calcified specimens could be cut thinner). Each cut was immediately placed on Kapton film and covered with Kapton adhesive tape. This created an airtight seal that prevented dehydration of the samples that can cause severe tissue warping. Samples were then flattened between two microscope slides and transported to the Australian Synchrotron in Melbourne.

### Scanning x-ray fluorescence imaging of vertebral sections

Samples were attached to polycarbonate frames at the Australian Synchrotron’s X-ray Fluorescence Microprobe^56^ using clear double-sided tape, with roughly 15–20 samples per frame. Smaller samples were scanned at 15 μm intervals (e.g., *S. albipunctata*) while larger samples were scanned at 25 μm intervals (e.g., *Charcarhinid* spp., *C. carcharias, S. zygaena*). Sampling intervals were chosen to balance scanning time and measurement sensitivity with the expected length scale of elemental variations. Per-pixel dwell times were typically 10 ms, and the scanning time for each frame was between 16 and 22 h, depending on the dwell, the samples, and scanned area. Elemental maps were created and viewed using GeoPIXE^57^, which uses a detailed specimen model to determine first-order depth-independent elemental concentrations. A series of x-ray fluorescence concentration standards (Micromatter) were measured at regular intervals through the experiment to control for experimental drift if present; none was observed, and so all data were processed using a single fit model.

The quantitative accuracy of elemental mapping relies on the uniform illumination of a narrow column through a specimen, and the measurement of characteristic x-ray fluorescence emitted from that column. The high incident x-ray energy (18.5 keV) ensured uniform illumination; however, the lower energy of the characteristic x-ray fluorescence can result in significant absorption of the x-ray fluorescence for various elements. The absorption length of calcium fluorescence (~3.7 keV) in water (a crude model for wet cartilage) is around 110 μm, whereas that of strontium (~14.1 keV) is 500 μm, leading to a strong dependence of the depth sensitivity on elemental species. The matrix correction applied by GeoPIXE corrects for this differential escape depth, assuming that the elemental distribution is uniform through the thickness. Here we have recorded elemental maps with the illumination closely aligned along the elemental bands so that this assumption is valid. Any sample misalignment would result in a blurring of the strontium distribution relative to calcium due to the increased depth penetration, and such is not observed in our elemental images.

To determine age, elemental lineouts were taken from data from the *corpus calcarum*, from the centre to the outer edge. Data were transformed using a moving six-sample mean to reduce noise. Sample results post-birth mark (defined as an angle change on the centrum face^9^) were counted for large peaks using a moving framework and a six-point moving average (more than 2.5% variation) in both the calcium and strontium spectrums.

### Sample microscope imaging

Traditional visual ageing was done on 0.6 mm sections of the vertebrae directly adjacent to the vertebra used for the SXFM analysis (thus in total, two vertebrae were used for each shark in this study, for a total of 12 vertebrae and 6 species). Vertebrae were manually cleaned with a scalpel and sectioned using an Isomet diamond saw. Excess tissue remaining after the sectioning was also removed with a scalpel. No chemicals were used when preparing these vertebrae to enable effective comparison with the SXFM samples. Vertebrae were observed in saltwater under a high-contrast binocular microscope immediately after sectioning. Images were taken via USB camera and saved with ImageJ software, where optimum lighting was applied to increase the contrast of growth bands. Images of the samples were then independently aged by two experienced observers after establishing band criteria for each species. A yearly increment consisted of one opaque and one translucent band^9^. Only vertebrae where the growth bands were well defined and visible were scored. Outside experts helped determine the band counts using similar criteria of the *C. carcharias, C. brevipenna* and *C. obscurus*, as the available samples were difficult to age. These experts aged the specimens blindly, with no knowledge of the sharks’ age or sex. To compare how well strontium and calcium peak counts matched the numbers of visual growth bands, data were analyzed using a multiple linear regression analysis with an alpha of 5%. All data analyses were done using Microsoft Excel and the Real Statistics data analysis tool pack.

## Additional Information

**How to cite this article**: Raoult, V. *et al*. Strontium mineralization of shark vertebrae. *Sci. Rep*. **6**, 29698; doi: 10.1038/srep29698 (2016).
